# Child and adolescent mental health services in Khartoum State, Sudan: a desktop situational analysis

**DOI:** 10.1186/s13034-024-00707-1

**Published:** 2024-02-03

**Authors:** Khalid A. Abdalhai, Stella Mokitimi, Petrus J. de Vries

**Affiliations:** 1 Department of Psychiatry, Faculty of Medicine, International University of Africa, Khartoum, Sudan; 2 Division of Child and Adolescent Psychiatry, University of Cape Town, Cape Town, South Africa

**Keywords:** Child, Adolescent, Mental health, Situational analysis, Khartoum, Sudan

## Abstract

**Background:**

Sudan is a Northeast African country, with 61.7% of its population under 24 years. With a large youth population and significant cultural and linguistic diversity, Sudan, like most low-income countries, has contributed minimal data to global child and adolescent mental health (CAMH) research. This study aimed to perform the first ever situational analysis of CAMH services and systems in Khartoum State, Sudan.

**Methods:**

The study focused on Khartoum state and covered the calendar years 2019 and 2020. Using the World Health Organization Assessment Instrument for Mental Health Systems (WHO-AIMS) version 2.2 adapted for CAMH, the study focused on the publically available data sources. Findings were described and presented in tables and figures using the WHO-AIMS template.

**Results:**

The situational analysis found no CAMH-specific policies, no separate budget for CAMH, and no supervising body for CAMH services in Khartoum. Three tertiary mental health hospitals provided CAMH services, all combined with adult mental health services. Essential medicines were available in all facilities, except methylphenidate available only in 3 central pharmacies. There was no free access to essential psychotropic drugs for children and adolescents except in emergency settings. Data about training to primary healthcare providers and the process of referral to specialized services were limited. A school mental health programme existed which provided early identification and management of CAMH problems in schools. The workforce was small and variable across all levels of care. No formal public health awareness campaigns and little evidence of formal intersectoral collaboration on CAMH were identified. A health information system existed, but no CAMH-specific items were reported. Among a handful of publications on CAMH, no national studies on CAMH were identified.

**Conclusions:**

This situational analysis represented the first systematic collation of data and information about CAMH in Sudan. Findings highlighted some areas of strength, but also many gaps in CAMH services and systems. We acknowledge the need to complement the desktop analysis with in-depth data collection with stakeholders across multiple levels, but hope that this will serve as a first step towards strengthening CAMH services in Sudan and other low-income countries.

## Background

Mental health is a key component of health. Unfortunately, the Africa region which has a large youth population and significant cultural and linguistic diversity, has contributed very limited data to global child & adolescent mental health (CAMH) research [1, 2]. Nevertheless, there is a growing awareness of the importance of CAMH, which has shaped global health initiatives over the past two decades [3]. A recent systematic review and meta-analysis showed considerable mental health problems among children and adolescents in sub-Saharan Africa (SSA), with 19.8% positive on screening tools, 14.3% meeting criteria for one or more psychopathology, and 9.5% diagnosed with clinical diagnostic instruments [4]. Despite these facts, CAMH services on the African continent are still underdeveloped, and CAMH research is highly lacking [2, 5–8]. In a study conducted by Davis and colleagues about CAMH in Africa, the authors identified significant gaps in CAMH-related policies, community participation, CAMH institutions, and interpersonal communication in relation to CAMH [9].

The World Health Organization (WHO) developed a framework to guide the evaluation and strengthening of mental health services globally [10]. The ‘healthcare pyramid’ framework proposed that tertiary and specialist services are costly and should be required by only a small portion of the population, while informal, community-based, and primary healthcare services (which can be provided at a relatively low cost) should be made available to a large proportion of the population [10].

**Fig. 1:**
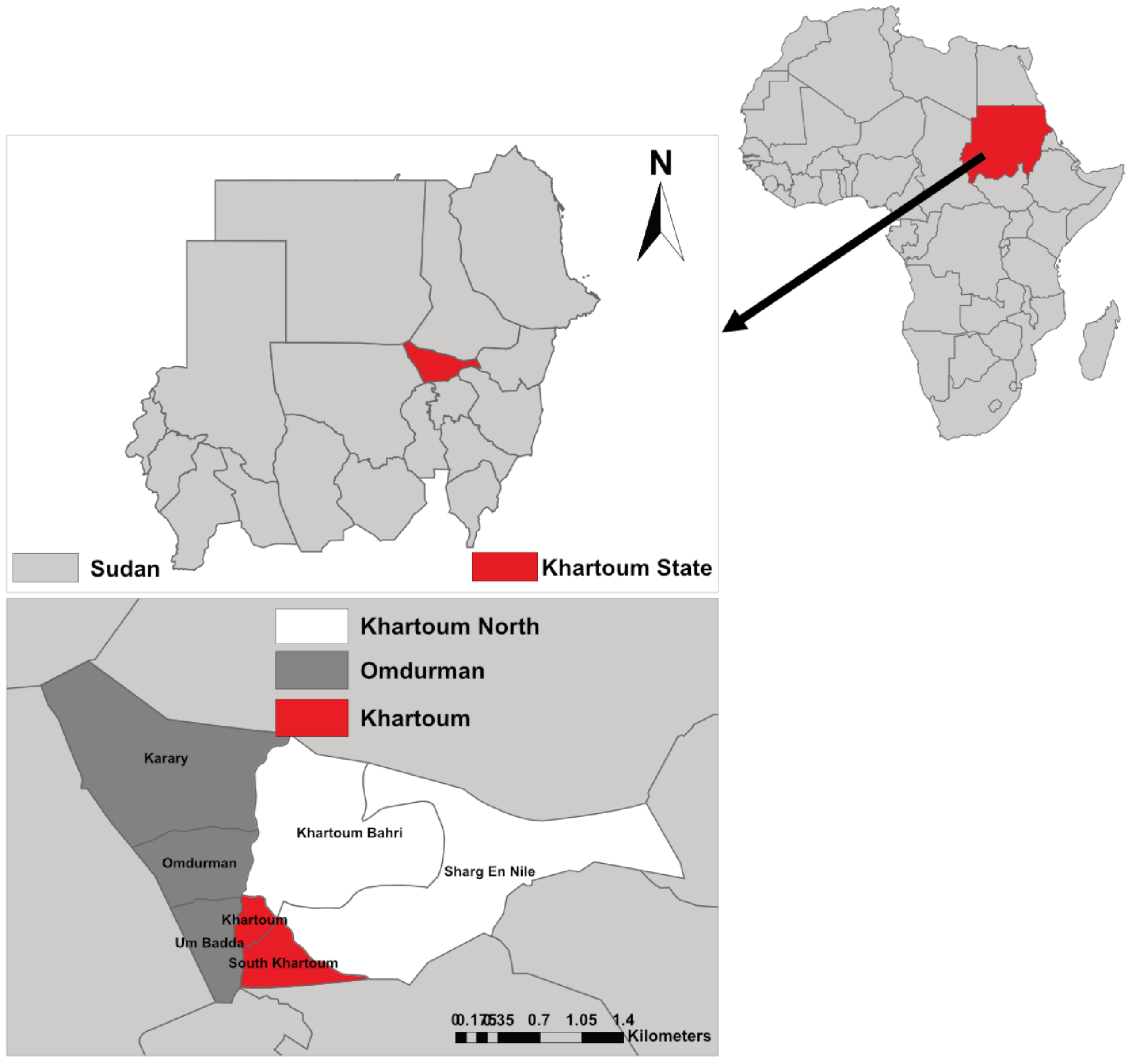
A map of Sudan and the Khartoum State

Sudan is a low-income African country in the Northeast part of Africa, bordered by seven countries - Egypt, Eritrea, Ethiopia, South Sudan, the Central African Republic, Chad, and Libya. It occupies 1.8 million km^2^ and has an estimated population of 38.6 million, of which 61.7% are under the age of 24 years [11]. Recent United Nations Population Fund data showed that about 39.5% of Sudanese people are between 0 and 14 years [12]. Two-thirds (66.7%) of the Sudan population live in rural settings, and almost half of the overall population (both urban and rural) live at or below the poverty threshold. The country was ranked 166 out of 187 countries across the world on the Human Development Index. The yearly GDP per capita in 2019 was around 2000 USD [12]. Sudan consists of 18 states (provinces), with Khartoum as the capital state [13] with an area of 22,000 km^2^. Official estimates in 2005 put the population of the capital city at 4.5 million, although unofficial estimates suggest a population over 7 million [14]. As shown in Fig. 1, Khartoum state is divided into seven localities (Khartoum, Jabal Awliya, Omdurman, Ombada, Karary, Bahry, and Sharq Enil).

## The mental health system in Sudan

The governmental health system in Sudan is managed at three levels: federal (one Federal Ministry of Health), state (18 states Ministries of Health), and district/ locality levels. The focus of these levels ranges from health policies and strategic planning to services implementation, management, and prevention [15]. As reported in the WHO Atlas of Mental Health, the absolute number of workers per 100,000 population varies enormously, from 11.9 psychiatrists per 100,000 population in high-income countries to fewer than 0.1 in low-income countries [8]. The WHO Atlas showed the number of general psychiatrists in Sudan to be 0.08 per 100 000, and the number of child psychiatrists 0.01 per 100 000 [16]. Osman and colleagues estimated the total number of psychiatrists in Sudan to be around 70 to 80 [17] with the majority (60–70) of them working in Khartoum state. It is not known how many psychiatrists work in rural parts of Khartoum or Sudan. Since 1990 specialization bodies have graduated 178 psychiatrists, of which 75% have left Sudan to work abroad. The average number of graduated per year is estimated to be 10 to 15 [17]. Sudan does not currently have the skill-set to train child & adolescent psychiatrists, and all of the existing two child and adolescent psychiatrists have trained on other countries, such as South Africa or the United Kingdom. The CAMH services are located only in Khartoum and Gezira states (both are middle states in Sudan). Within the Khartoum state, CAMH services are theoretically provided at all health service levels [17, 18].

The Sudan Mental Health Policy [19] was published with the support of the WHO in 2009, with the following components: (1) the development of a mental health component in primary healthcare, (2) scaling up human resources, (3) the involvement of patients and their families, (4) strengthening advocacy, (5) promotion of the human rights protection of patients, (6) equity and access to mental healthcare services across different groups, (7) quality improvement, (8) financing and (9) establishing monitoring systems [20].

Postgraduate training in psychiatry is overseen by the Sudan Medical Specialization Board (SMSB), established in 1995 to produce highly qualified medical specialists and subspecialists. The four-year training programme in general psychiatry includes training in different specialities in psychiatry plus a 3-months rotation in adult neurology. In addition, all candidates should spend three to six months on child and adolescent psychiatry during the training period, focusing on outpatient cases. While the country has witnessed a significant increase in the number of mental health professionals, over the past two decades, until 2019, 178 graduates have become specialists in psychiatry, 75% of whom have since emigrated, primarily to work in the Gulf States [18]. Unfortunately, there are no available preliminary data regarding the number of psychologists, psychiatric nurses, and social workers in postgraduate programmes [21].

In the context of the limited knowledge-base about mental health and psychiatric services in Sudan, CAMH knowledge are even more limited. To our knowledge, no national epidemiological study on CAMH has been performed to date in Sudan, and few ‘data’ publications on CAMH have been published. In one study the prevalence of depression and anxiety among school-age children was reported to be 12% [22]. In a study of adolescents in a correctional facility in Khartoum state, psychiatric disorders were reported to be high (60%), with conduct disorder the most common (47.9%), followed by anxiety disorders (31.1%) and major depressive disorder (14.6%) [23]. The WHO reported an all-ages suicide rate of 8.1. per 100,000 in Sudan [24], but unfortunately, these data were not disaggregated to show suicide rates for under-18-year-olds. It is clear that these limited data provide snapshots from different and potentially biased samples, thus making it very difficult to have an accurate and data-driven picture of the mental health needs of children and adolescents in the country.

Based on a meta-analysis of CAMH disorders that show up to 20% of the full range of child & adolescent psychopathologies globally [25], Sudan would (at a conservative estimate of a 10% prevalence) have ~ 2.38 million children, adolescents and young adults under the age of 24 with a diagnosable and treatable mental health disorder. However, despite these very high numbers indicating very significant levels of need for CAMH services and systems of care in the country, almost nothing is known about the basic building blocks and components of CAMH services and systems in Sudan.

## Situational analysis of CAMH services and systems (CAMHSS)

The WHO defined situational analysis in the health sector as “an assessment of the current health situation … fundamental to designing and updating national policies, strategies, and plans” [26]. There are many other definitions for situational analysis, but they share the same concepts. Performing situational analyses in the health sector is often an ongoing process. It aims to (1) assess the current health sector situation, including strengths, weaknesses, opportunities, and threats, (2) provide evidence for responding to health sector needs and expectations of the population, and (3) provide evidence for formulating future strategic plans for the health sector [27].

The WHO defined health systems as “all the organizations, institutions, resources and people whose primary purpose is to improve health” [28] and proposed six building blocks that can also be applied to mental health: (1) service delivery, (2) health workforce, (3) health information systems, (4) access to essential medicines, (5) financing, and (6) leadership/governance [28, 29]. Therefore, a comprehensive evaluation of a health system requires focusing on all the domains mentioned above.

Given the limited knowledge about CAMHSS in Sudan, this study set out to perform a desktop situational analysis of CAMH services and systems in Khartoum state, Sudan.

## Methods

### Study design

This was a desktop cross-sectional descriptive situational analysis performed using a modified version of the World Health Organization Assessment Instrument for Mental Health Systems (WHO-AIMS) version 2.2 of 2005, to collect data about CAMHSS in Khartoum. This modified version adapted from the WHO-AIMS is, to our knowledge, the only published assessment tool for situational analysis of CAMH services [7].

### The context and boundaries of the study

The study was performed in the national capital of Sudan, Khartoum state, and focused on a period of 24 months (from the 1st of January 2019 to the 31st of December 2020). This period was selected because it represented a transition between the previous governing regime and the current transitional government.

### Data collection

The modified version of the WHO-AIMS, version 2.2 of 2005 was used for data collection. As outlined by Mokitimi and colleagues [7], the WHO-AIMS was designed for general mental health systems, and only one CAMH-related item was included in the original tool. Mokitimi and colleagues adapted the WHO-AIMS for CAMH data collection by (a) selecting relevant items from the tool and (b) adapting the descriptions to be CAMH-specific. We, therefore, opted to use the Mokitimi adaptation for data collection here [7]. Items selected for this study are shown in Table 1. The information about individual data items shown in Table 1, was collected by using publically available data, and the gaps were filled by direct communication with relevant data sources. The researcher engaged with key stakeholders telephonically and by email to obtain information to fill in the gaps, and verify the information already obtained.

**Table 1:** Items selected for the desktop situational analysis of CAMH services in Khartoum State, Sudan and related data sources

WHO-AIMS Domains	Potential Data Sources
**1. Policy and legislative framework**	
1.1 CAMH Policies, plans, and legislations (B1, B3, B4) 1.2 Human rights legislation relevant to children and adolescents (B5) 1.3. Financing: Expenditure on child and adolescent mental health services by the Provincial Department of Health (B6)	The Head of the Mental Health Council in the Federal Ministry of Health Child and Family Law The Mental Health Act of Sudan The Finance Division at the Khartoum Ministry of Health
**2. Child and adolescent mental health services**	
2.1. Existence and functions of a regional CAMH authority (B9) 2.2. Organization of CAMHS in terms of catchment areas (B10) 2.3. Outpatient services: Availability of CAMH outpatient facilities, and number/proportion of children and adolescents treated for mental health problems through outpatient facilities at primary, secondary, and tertiary levels of care (B11, B12, B13) 2.4. Inpatient services: Availability of CAMH inpatient facilities and number/proportion of children and adolescents treated (B15, B16, B17) 2.5. Availability of CAMH day patient facilities, community residential facilities, forensic facilities, or CAMH hospitals (B14, B18, B19, B25) 2.6. Interventions (Medications): Psychotropic medicines appropriate for children and adolescents are included on the essential medicines list, free access to essential psychotropic medicines, and availability of medicines in outpatient and inpatient settings at secondary and tertiary levels of care (B2, B8, B28, B29) 2.7. Interventions (Psychosocial): Access to psychosocial interventions in outpatient and inpatient settings at secondary and tertiary levels of care (B26, B27)	The Hospital Directorate in Khartoum state Ministry of Health The Directorate of Therapeutic Medicine in Khartoum state Ministry of Health The Directorate of Preventive Medicine in Khartoum state Ministry of Health The Head of the Psychiatry Consultancy Board in the Federal Ministry of Health Psychiatric Hospital Directors Sudan National Essential Medicines List 2014 (or more recent versions if available)
**3. Child and adolescent mental health in primary healthcare (PHC)**	
3.1. Refresher training in CAMH provided to primary health care (PHC) doctors, nurses or other staff and interaction of PHC with specialist CAMHS (B31-B35) 3.2. Availability of medicines and psychosocial interventions in PHC facilities (B27, B33)	The Directorate of Primary Health Care, Khartoum state Ministry of Health The Director of the School Mental Health Programme at Khartoum state Ministry of Health Sudan National Essential Medicines List 2017
**4. Human resources**	
4.1. Human resources in CAMH services (B38-B41)	Human Resources Section, Khartoum State Ministry of Health Hospital Directors
**5. Public education and links with other sectors**	
5.1. Public education and awareness campaigns about CAMH (B47)	Khartoum State Ministry of Health, Directorate of Health Education and Awareness WHO office of Sudan
**6. Monitoring and research**	
6.1. Monitoring CAMH services (B52, B53)	Directorate of Hospitals, Khartoum State Ministry of Health
- Data transmission from mental health facilities (B52) - Report on mental health services by the government health department (B53)	The Head of the Psychiatry Consultancy Board in the Federal Ministry of Health
6.2. Research in CAMH (B54)	Sudan Medical Specialization Board University of Khartoum library Online search for peer-reviewed publications.

* The numbered letters (e.g., B1, B2, B3….etc.) represent the item codes for subdomains from the original brief version of the WHO-AIMS 2.2

### Data analysis

Data were analysed using descriptive statistics supported by the WHO-AIMS version 2.2 of the 2005 template and presented in tables and figures with a relevant description of each domain.

### Research ethics

The study protocol was approved by the Faculty of Health Sciences at the University of Cape Town (HREC026/2022) where the first author was a registered MPhil student.

## Results

The desktop situational analysis findings using the adapted variables from the WHO-AIMS (as shown in Table 1) are presented here. Results are presented by domain using the WHO-AIMS summary template [30].

### WHO-AIMS domain 1: policy and legislative framework

#### Policies, plans and legislations

We could not identify any CAMH policy, legislation, or plans at the national or state level [19]. The Sudan National Mental Health Policy was last revised in 2009 in collaboration with the WHO and covered the following components: (1) development of mental health services in primary healthcare, (2) scaling up human resources, (3) involvement of patients and their families, (4) strengthening advocacy, (5) promotion of the human rights protection of patients, (6) equity and access to mental healthcare services across different groups, (7) quality improvement, (8) financing and (9) establishing monitoring systems [20]. The policy was developed by a committee that included representatives from different Sudanese universities, Federal and States Ministries of Health, and the Ministry of Social Affairs. There were no stand-alone or CAMH-specific policies or plans identified. However, in the Sudan Mental Health Policy, CAMH was mentioned under specialized programmes that need to be developed together with addiction and old age psychiatry. Despite the absence of legislation specific to CAMH, the Children’s Act (2010), released by the National Council for Child Welfare under the supervision of the Ministry of Justice in 2010, represented legislation relevant to children under the age of 18 years and laid the foundation for child protection and welfare in Sudan. The act was considered important legislation that addressed the issues of children’s need for protection, care, safety, and justice; and defined the child and the age of criminal responsibility. The act did not specify how these concepts should be applied [31].

#### Human right relevant to children and adolescents

Despite the human rights domain being mentioned in the Sudan Mental Health Policy [19], no human rights review body existed in Sudan during this situational analysis. At the country level, the Psychiatry Consultancy Board (PCB), a body consisting of senior mental health professionals in the county and directly related to the Sudanese Federal Ministry of Health, provided advice to the government related to human rights, legislation, service coordination, and planning. However, the PCB was not involved in any executive decision-making [Personal communication, Dr Salah Haroun, Previous head of the PCB, 29 Nov 2021]. In Khartoum, there was no reported review or inspection of the human rights protection of children and adolescents during the situational analysis period, either in mental health facilities or in community-based services. Furthermore, we found no evidence that mental hospital staff or staff working in inpatient psychiatric units or community residential facilities had received teaching or training sessions on the human rights protection of patients during the study period [Personal communication, Medical directors of the psychiatric facilities in Khartoum, 13 December 2021].

#### Expenditure on CAMH services in Khartoum

The situational analysis was not able to identify any data related to the financing of mental health or CAMH in Khartoum. About 6.5% of Sudan’s Gross Domestic Product (% GDP) and 8.2% of the general government expenditure on health in general. The out-of-pocket share was about 70% (US$84.0 per capita), while the general government health expenditure represents only 22.3% (US$26.9 per capita) [32].

### WHO-AIMS domain 2: child and adolescent mental health services

#### Existence and functions of a regional CAMH authority

As outlined under domain 1, a PCB existed at Federal Level, but no health authority in the country was devoted to overseeing CAMH. In practice, the directors of the psychiatric hospitals functioned as leading authorities for mental health in their facilities [Personal communication, Previous head of the PCB, Dr Salah Haroun, 29 Nov 2021]. However, these facilities typically did not include many children and adolescent mental health specialists.

#### Organization of CAMH services in terms of catchment areas

The mental health service provision in Khartoum state was under the umbrella of the directorates of curative medicine [19]. Theoretically, mental health services in Khartoum were organized in terms of geographical (i.e., catchment areas) service areas. However, the structure was strongly centralized [18]. Khartoum state was divided into three major cities, Khartoum, Omdurman, and Bahri. Each city was further divided into localities, with a total of seven localities in the whole of Khartoum state.

#### Availability of CAMH outpatient facilities, and number/proportion of children and adolescents treated for mental health problems through outpatient facilities at primary, secondary, and tertiary levels of care

We were unable to find any publicly available data about CAMH services in Khartoum. However, on the ground, CAMH services were available in three specialist mental and paediatric health facilities in Khartoum, distributed in two cities of Khartoum state (two in Omdurman, one in Bahari). The first service was a weekly outpatient service for children with mental health problems established as a package of care through the Department of Paediatrics at the Military hospital in 1998 and supervised by a child psychiatrist. The second service was also an outpatient and inpatient service for children and adolescents with mental health problems based in a Taha Baashar psychiatric hospital in Bahri, established in 2011 and supervised by a child psychiatrist. Inpatient care for children was provided in an adult mental health ward. The third service for children and adolescents with mental health problems was in the Alzahra centre (a mother-and-child unit) in the Eltigani Elmahi Psychiatric hospital in Omdurman. This centre provided outpatient (3 days per week) and inpatient services for children and adolescents with mental health problems (in a separate two-bed ward). The centre was established in 2015 and was supervised by a general psychiatrist interested in childhood psychiatric disorders. According to data from health facilities that ran CAMH services, 980 children and adolescents were seen during the study period from January 2019 to December 2020. Out of all children and adolescents treated as outpatients in these facilities, 27.55% (*N* = 270) were female. Further details are shown in Table 2.

**Table 2:** The number and percentage of children and adolescents seen at the different mental health outpatient facilities during the study period in Khartoum state

		Jan - December 2019	Jan – December 2020
The Military Hospital	Total number	276	87
	Sex	Females = 80 (29%)	Females = 23 (26.4%)
		Males = 169 (71.1%)	Males = 64 (73.6%)
Taha Baashar Hospital	Total number	92	77
	Sex	Females = 43 (46.7%)	Females = 34 (44.2%)
		Males = 49 (53.3%)	Males = 43 (55.9%)
El Tigani Elmahi Hospital (Alzahra Centre)	Total number	218	138
	Sex	Females = 76 (34.7%)	Females = 23 (16.7%)
		Males = 142 (65.3%)	Males = 115 (83.3%)

The data from the specialist facilities that provided CAMH services revealed the most frequently diagnosed disorders (from the most to the least frequent) in the outpatient clinics of Khartoum during the study period were: (1) unspecified psychological and behavioural conditions, (2) intellectual disability, (3) epilepsy, (4) attention deficit hyperactivity disorder, (5) schizophrenia and related psychotic disorders, (6) autism spectrum disorder, and (7) disruptive behaviour disorders. These diagnoses were based on the outpatient facilities registry in the three hospitals that provided mental disorders services for children and adolescents. The average number of hospital/clinic contacts per user was not available. There was no organized follow-up care in the community for the individuals seen at outpatient facilities that provide CAMH services. All three outpatient facilities had access to non-medical treatments such as occupational therapy, speech and language therapy, and psychological therapies. There were no available data about the type of psychological intervention specific to children and adolescents with mental health problems. Furthermore, whenever specialized psychotherapies (i.e., psychotherapy for OCD, Tic disorder, behavioural interventions for autism etc.) were needed, patients and their families were referred to private clinics, which mostly provided CBT-informed therapies.

#### Availability of CAMH inpatient facilities and number/proportion of children and adolescents treated

At the time of the situational analysis, there were three general adult psychiatric hospitals in Khartoum, with a total of 146 beds (Male = 94; Female = 52), representing 2.9 beds per 100,000 of the Khartoum population [17]. All of these facilities were organizationally integrated with mental health outpatient facilities. No beds in general psychiatry hospitals were dedicated only to children or adolescents during the study period from January 2019 to December 2020. Therefore, if admission was needed for a pre-adolescent (child), admission was to the female ward. Adolescents were admitted to a male ward. It is also important to mention that the admission of children and adolescents to these facilities was voluntary. Based on data from the registry of facilities that provided services for CAMH services, children and adolescents admitted to mental hospitals were primarily from four diagnostic groups: (1) unspecified psychological and behavioural disorders, (2) psychiatric disorders due to underlying medical conditions, (3) schizophrenia and related psychotic disorders, and (4) mood disorders. The average number of days spent in mental hospitals was not stated. However, informal data from practising clinicians working in CAMH facilities suggested that the admission period typically lasted between 7 and 14 days [Personal communication, Dr Emad Elsunni, Dr Mohja Ibrahim, and Dr Lubaba Abdalla, psychiatrists at Taha Baashar and Eltigani Elmahi Psychiatric Hospitals, 12 December 2021]. There were no CAMH day patient facilities, community residential facilities, forensic facilities, or CAMH hospitals in Khartoum state during the study period. The details of inpatients admitted to the facilities that provided CAMH services were available in Taha Baashar hospital, as presented in Table 3. Unfortunately, the number and percentages of inpatients below 18 years admitted with a mental health problem in the Military and Eltigani Elmahi hospitals could not be identified.

**Table 3:** The number of children and adolescents treated as inpatients at Taha Baashar Hospital in Khartoum in 2019 and 2020

Age group	Number and gender	Jan-December 2019	Jan-December 2020
Age 0–4 years	Total number	10	9
	Sex	Females = 5 (50%)	Females = 4 (44.4%)
		Males = 5 (50%)	Males = 5 (55.6%)
Age 5–14 years	Total number	198	214
	Sex	Females = 75 (37.9%)	Females = 101 (47.2%)
		Males = 123 (62.1%)	Males = 113 (52.8%)
Age 15–24 years	Total number	2257	2379
	Sex	Females = 807 (35.7%)	Females = 877 (34.7%)
		Males = 1450 (64.3%)	Males = 1502 (65.3%)

#### Interventions (Medications): Psychotropic medicines appropriate for children and adolescents are included on the essential medicines list, and free access to essential psychotropic medicines, and availability of medicines in outpatient and inpatient settings at secondary and tertiary levels of care

Regarding the availability of psychotropic medicines, the supply in the country was interrupted in 2019 and 2020 due to a combination of the COVID-19 pandemic, an unstable economy, and the political situation in the country [33]. Despite these challenges, facilities that provide mental health services for children and adolescents had at least one psychotropic medicine of each therapeutic class relevant to children and adolescents (antipsychotic, antidepressant, mood stabilizer, anxiolytic, and antiepileptic medicines). However, medications used for ADHD (i.e., methylphenidate) were classified in the Sudan Essential Medicines List in the category of drugs prescribed by specialists service. Hence it was available only in three pharmacies in Khartoum state [Personal communication, Dr Samah, Pharmacist at Taha Baashar Hospital, 13 December 2021]. Health insurance was available and provided by different insurance companies, however, the percentage of children and adolescents covered was not clear.

According to the National Essential Medicines List (2014), three medications were listed in the child psychiatry category. These included atomoxetine (a non-stimulant medication for ADHD), methylphenidate (a stimulant medication for ADHD), and clonazepam (a benzodiazepine). There were no antidepressant, anti-anxiety, or antipsychotic medications for children included in the essential medicines list category during the study period. However, other medications used in CAMH were included under general psychotropic medicines (e.g., risperidone, clozapine, fluoxetine, sertraline, imipramine, valproic acid, lithium, carbamazepine and lorazepam). Methylphenidate was a controlled medication that was classified in the list of essential medicines as “medicines used by specialized centres and units in some designated hospitals” and was only available in National Medical Supplies Fund pharmacies [34]. The Sudan Nation Supply Fund was established in 1935 as a central drug store affiliated with the Federal Ministry of Health. The main objectives were to increase coverage of essential and affordable medicines and prevention of the distribution of medicines from unreliable sources [34].

### WHO-AIMS domain 3: child and adolescent mental health in primary healthcare

#### Refresher training in CAMH provided to primary health care (PHC) doctors, nurses or other staff and interaction of PHC with specialist CAMHS

**Fig. 2:**
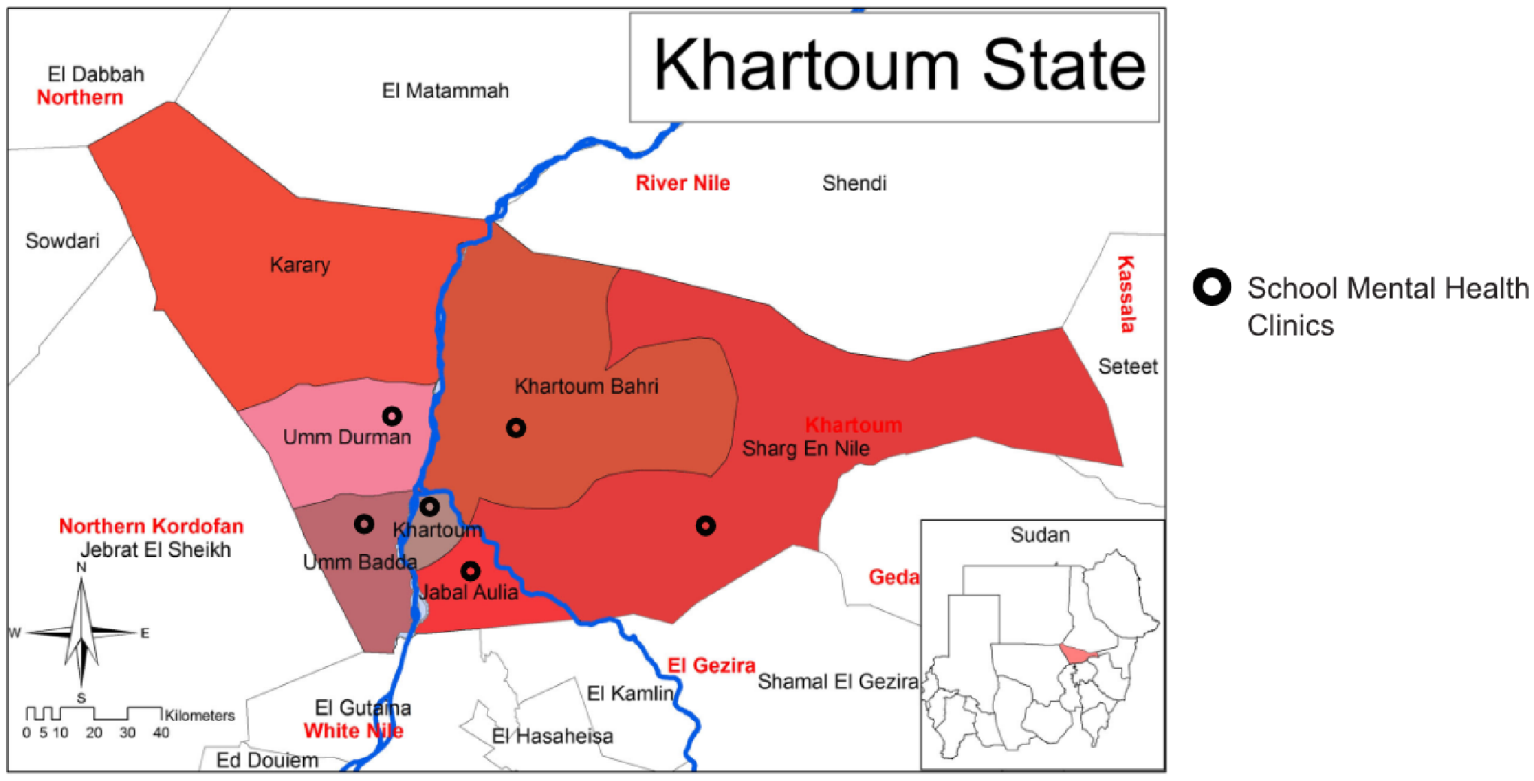
The figure shows the distribution of the school mental health clinics in Khartoum state

During the study period, the team could not identify information about CAMH at the primary care (PHC) level in Khartoum. However, in a WHO report on mental health in Sudan, clinics in primary care were described as both physician-based and non-physician-based [20]. The majority (> 80%) had no assessment or treatment protocols for key mental health conditions available. They could diagnose and treat some psychiatric disorders in adults (e.g., depression, bipolar disorders, psychotic disorders). No data were available regarding mental health problems in children and adolescents [20]. The majority of the primary health care clinics (51–80%) made at least one monthly referral to a mental health professional. There was no mention of a referral process or whether it included a referral to specialized facilities that provide mental health services to children and adolescents. There was no clear communication process between primary care physicians and mental health professionals regarding individuals with mental disorders, including children and adolescents [20]. Interestingly, a School Mental Health Programme, a part of the school health programme, was represented in eight primary health care clinics geographically distributed in the Khartoum state localities (see Fig. 2). The programme was supervised by the Khartoum Ministry of Health (the School Health Directorate) in collaboration with the Khartoum Ministry of Education. The school mental health clinics, established in 1998, provided mental health services to school-aged children. Unfortunately, we were not able to identify any specific numbers for children and adolescents seen during the study period. The programme included a package of early detection, intervention, management, and follow-up for school-age children with various psychiatric disorders. The process started with a mobile team (that included medical doctors, psychologists, dental care specialists, audiologists, and nutritionists), providing basic health education (including mental health) to teachers and students in all the government schools in Khartoum. Then a focal person (either a teacher or an educational psychologist) would then be allocated to report children at risk and children with symptoms of mental health problems. After that, the caregiver of a child with a mental health problem would be given a referral form to be seen in the nearest school mental health clinic. If further referral to a higher level of specialized services was needed, the process was unclear. In the SMH clinics, the staff included mainly psychologists. No psychiatric doctors were included since 2018 [Personal communication, Mrs Asma, coordinator of the SMH program, 12 January 2022]. Before 2019, psychiatry specialists used to see children with mental health problems twice weekly at SMH clinics. Most psychiatric doctors in the SMH programme were part-timers and resigned because of low payment [Personal communication, Dr Safa Elsarrg and Dr Bahja Hamed, psychiatrists who worked in SMH clinics, 10 December 2021].

The situational analysis also identified centres for traditional healing in the community, not regulated by the Ministry of Health [20]. They provided religious, spiritual, and cultural healing for their client, including children and adolescents, without basic mental health training. We were not able to find data about the number of clients, types of interventions, outcomes and feedback about these centres.

We were not able to find data regarding refresher training on CAMH for doctors, nurses, or other staff provided at the primary healthcare level in Khartoum state during the study period. The last time practitioners at the primary care level received a comprehensive training course in mental health was in 2016 and organized by the Khartoum Ministry of Health and included primary care trainees. The training programme included topics on child and adolescent mental health [Personal communication, Dr Amal Eltigani, Psychiatrist, the Director of Eltigani Elmahi Hospital and mhGAP trainer, 10 February 2022].

#### Availability of medicines and psychosocial interventions in PHC facilities

In Sudan, medically-qualified and non-medically qualified primary health care workers (referred to as ‘medical assistants’) can prescribe basic psychotropic medications to children and adolescents that are classified under categories AA, A, and B in the essential medicines list (i.e., medicines that used in health facilities run by community worker, medical assistants, and medical doctors) [35]. This included imipramine, amitriptyline, clomipramine, carbamazepine, and diazepam. The use of psychotropic medications for children with mental health problems by primary care providers was not clearly stated. Stimulants and non-stimulant medications for ADHD were only prescribed in a specialist services/ unit. In real-life practice, primary health care providers could prescribe a wide range of psychotropic medications to children and adolescents (e.g., fluoxetine, sertraline, risperidone, and olanzapine) without restrictions, except for stimulant medications, as outlined earlier. As for the availability of psychotropic medicines, most physician-based PHC centres had at least one psychotropic medicine of each therapeutic category (antipsychotic, antidepressant, mood stabilizer, anxiolytic, and antiepileptic) either in the facility or a nearby private pharmacy (which worked independently from the government sector). Medications were not provided free of charge except in the case of psychiatric emergencies, and the cost of the cheapest antipsychotic medication represented approximately 27% of the minimum daily wage, and the cost of the cheapest antidepressant medication ~ 18% of the minimum daily wage [20, 35]. There were no differences in the prices of psychotropic medication between the primary and specialist mental health services for children and adolescents.

### WHO-AIMS domain 4: human resources

#### Human resources in CAMH services

The WHO estimated the total number of human resources working in mental health facilities in Sudan to be 0.92 per 100,000 population [20]. The total number of workforces in the mental health facilities that provide care for children and adolescents in Khartoum was estimated to be 0.1 per 100,000 population during the study period. No formal human resource data were available for CAMH during the situational analysis period. However, as shown in Table 4, informal data suggested that 26 mental health professionals were working in facilities that provide services for children and adolescents [Personal communication from the three Psychiatric Hospital Directors and the coordinator of the SMH programme, 23 December 2021]. The two child psychiatrists worked both for government-administered CAMH facilities and in the private sector. The psychologists, social workers, and nurses worked for government-administered mental health facilities, either exclusively or also in other sectors.

**Table 4:** Informal estimate of the number and specialities of the mental health workforce who provided services for children and adolescents in Khartoum in 2019 and 2020

Professionals	Number
Child and adolescent psychiatrists	2
Psychiatrists	3
Psychiatry trainees	3
Psychiatric nurses	1
Psychologists	13
Social workers	4
Occupational therapists	0
Speech and language therapist	0

### WHO-AIMS domain 5: public education and links with other sectors

#### Public education and awareness campaigns about CAMH

There were no coordinated bodies at the Federal and State Ministries of Health to oversee public education and awareness-raising campaigns specific to CAMH in Khartoum state during the study period. As a result, we were not able to identify any awareness-raising programmes specific to CAMH in Khartoum state during the study period. However, different non-governmental organisations (NGOs), professional associations, private trusts/foundations, and international agencies have promoted public education and awareness campaigns during the study period [20]. These campaigns targeted the general population, children, adolescents, women, and trauma survivors, e.g. gender-based violence.

Regarding the link and the collaboration between Health and other sectors in Khartoum, there were formal collaborations with the departments/agencies responsible for primary health care, child and family protection, education, and social affairs [35]. However, as stated earlier, the link was poorly defined.

### WHO-AIMS domain 6: monitoring and research

#### Data transmission from mental health facilities and Reports on mental health services by the government health department

The Sudanese Health Information System (HIS) was one of the first information systems in the region [15]. It involved data collection, processing, analysis, and dissemination. Data in the health sector in Sudan is collected by individual health facilities and communicated to the State and Federal Ministries of Health. The information obtained was used to produce periodic reports, make decisions, allocate resources, and monitor the plans and strategies [36]. Unfortunately, the HIS had various weaknesses in which information collected was not pooled into the HIS, and some facilities collected and used data for their activities and then stopped without disseminating their findings [37]. In addition, there was an overall limited capacity for the analysis, utilization, and dissemination of data and findings [38]. There was no formally defined list of individual data items related to CAMH problems to be collected by all mental health facilities, as evident by the type of data from different facilities [Personal communication from the Head of HIS Department at the Khartoum Ministry of Health, 17 January 2022]. Instead, different mental health facilities registered different data related to CAMH (as shown earlier in Tables 3 and 4). These included the number of outpatient clinics per week, the number of patients seen in the facilities, diagnoses, and age ranges [Data department in Bashaar, Eltigani Elmahi, and the Military hospitals, 2021]. Furthermore, no formal reports were produced using the data transmitted to the Government Health Department [Personal communication from the Head of HIS Department at the Khartoum Ministry of Health, 17 January 2022].

#### Research in CAMH

In order to generate an overview of research relevant to child and adolescent mental health in Khartoum, we performed a data search for all relevant publications over ten years, including the two years of this situational analysis (2010–2020). Anticipating a low yield of peer-reviewed literature, we performed a broad online search on PubMed and Google scholar using “child and adolescent,” “mental health,” “Khartoum state,” and “Sudan” as keywords. In addition, we asked local mental health experts (psychiatric doctors and psychologists) working in Khartoum hospitals to identify any potentially relevant publications in the peer-reviewed and grey literature (including dissertations, reports, and non-peer-reviewed journals). As a result, a total of 11 articles were identified, as shown and described by study themes in Table 5. Six of the identified articles were about trauma and stress-related disorders, two were about neurodevelopmental disorders and disruptive behaviours, and one was about CAMH training in the middle-east region [20, 22, 38–46]. Furthermore, 90% (*N* = 10) of the identified research were conducted by Sudanese researchers.

**Table 5:** Research on CAMH in Khartoum state, Sudan between 2010–2020

Research theme	Research Topic	Reference (First author, journal, year)
Neurodevelopmental disorders	The prevalence and factors affecting attention deficit hyperactivity disorder among school children in Khartoum State [39]	Osman, A. M., *Sudanese Journal of Paediatrics*, 2015
	Psychosocial Impacts of Mentally Retarded Children on Parents in Sudan [40]	Shabo, F. H., *Sudan Journal of Medical Sciences*, 2011
Training and CAMH systems	Child and adolescent psychiatry training and services in the Middle East region: a current status assessment [21]	Clausen, C. E., *European Child & Adolescent Psychiatry*, 2019
Trauma and stress-related conditions	Child sexual abuse presenting to police centres in Khartoum-Sudan; pattern and victim-associated factors [41]	Elhassan, N. M., *MOJ Clinical & Medical Case Reports*, 2016
	Child Sexual Abuse Khartoum, Sudan: Pattern and Offender Associative Factors [42]	Abunaib, S., *EC Clinical And Medical Case Reports*, 2018
	Cultural, psychological and social dimensions of children in a situation of trafficking [43]	Elhassan, N. M., *The Arab Journal of the Social*, 2015
	The school environment and their impact on the creation of attitudes and future trends of violence (in the Sudanese universities) [44]	Elhassan, N. M., *The Arab Journal of the Social Sciences*, 2015
	Effect of Corporal Punishment on Sudanese Pupils from Parent’s Perspectives - Part 1 and Part 2 [45]	Elhassan, N. M., *MOJ Clinical & Medical Case Reports*, 2016
	Psychological impact on child soldiers in Sudan [46]	Elshiekh, A., *Sudanese Journal of Psychiatry*, 2011
Disruptive behaviours and related disorders	Research on the prevalence of conduct disorders among primary school pupils in Khartoum-Sudan [47]	Humaida, I. A. I., *Health*, 2012
	The nature and prevalence of psychiatric disorders in a Sudanese juvenile correctional facility [23]	Ali, A. S. *Sudanese Journal of Paediatrics*, 2016

## Discussion

This study set out to perform a desktop situational analysis of CAMH services and systems in Khartoum state, Sudan for the years 2019 and 2020 by assessing the basic building blocks of mental health systems using a modified version of the WHO-AIMS [7]. It was, to our knowledge, the first-ever attempt to describe CAMH services and systems in Sudan in a systematic way. Unfortunately, as anticipated, very limited CAMHSS data were available in the public domain, and we had to depend on the personal communications of key stakeholders in the health and education system in the State. However, this finding is very much in keeping with the literature which identifies CAMH as a largely neglected area within the mental health field [9, 30, 48]. Below we will summarise key findings from each of the WHO-AIMS building blocks and highlight gaps in knowledge about CAMH services and systems in Khartoum State.

There was no CAMH policy in Sudan, and legislation related to children and adolescents with mental health problems was lacking and poorly represented in existing documents [35]. There is therefore an urgent need to develop effective and practical national CAMH policies to outline service and system needs, and legislations that would address the protection of the rights of children and adolescents with mental health problems. We were not able to find evidence of the involvement of children, adolescents and/or families in the development, monitoring or improvement of services. This would be a fundamentally important component to add in practice to ensure effective collaboration between policymakers and stakeholders (e.g., ministers of health, organizations, families, and service users). There is also a need to disseminate legislation documents (i.e., Children’s Act, 2010) to professionals working in CAMH and related sectors to be put into practice. It was also unclear how mental health, including CAMH, is financed and supported by the government, representing another clear knowledge gap in this WHO-AIMS domain.

The CAMH services in Khartoum state were not well-defined and were poorly developed. This has unfortunately been the case in low-income countries for more than 40 years [2]. Clausen and colleagues studied the CAMH services and training in the middle east region and highlighted the deficiency in mental health professionals to treat children with mental health problems, reflecting the reality of insufficient specialists in LMICs [21]. The challenge of rebuilding and strengthening the existing CAMH systems and services can be reduced by improving access to services, reducing the stigma associated with mental disorders, and developing hospital-based specialist outpatient and inpatient CAMH services that are separate from adult health services [49, 50]. On the other hand, the shortage of qualified mental health professionals working in facilities that run services for children and adolescents represents another significant challenge (domain 4 of the WHO-AIMS). Lund and colleagues studied human resources and the cost required for improving the shortage of CAMH in South Africa by developing a model for that. They calculated that the minimum requirements per 100,000 population, the minimum coverage of full-time staff would need to be 5.8 in primary healthcare facilities, 0.6 in general hospital outpatient departments, 0.1 in general hospital inpatient facilities, 1.1 in specialist CAMHS outpatient departments, 0.6 in specialist CAMHS inpatient facilities, 0.5 in specialist CAMHS day services, and 0.8 in regional CAMHS teams [51]. However, it is clear that significant and sustained action over many decades will be required to create a CAMH specialist workforce to meet the needs of the population. Therefore, there is a clear need in Sudan also to consider how to use collaborative efforts to solve the problem of the shortage of health professionals and consider how to integrate CAMH services into paediatrics and general health programmes [52]. Such efforts would also need to establish clear referral pathways between different services and levels of care within the Khartoum health services. The study also found a reduction in the number of patients in 2020 in comparison to 2019. This was presumably attributable to the COVID-19 pandemic which brought lockdowns across most parts of the world, thus impeding access to healthcare.

The link between different mental health care levels in Khartoum was not well established, and we found little evidence of regular CAMH training for primary care staff. Overloaded services, shortage of human and financial resources, and low recognition of the importance of CAMH could lead to low motivation for primary healthcare workers to provide CAMHS [48]. Therefore, there is a need to establish training programmes in child & adolescent mental health for all professionals working at primary healthcare (PHC) level. At the PHC level, the training may target prevention, early detection, reporting, and management of CAMH problems. A study by Akol and colleagues in Uganda evaluated the effect of PHC provider mhGAP training on the identification of CAMH disorders. They concluded that mhGAP CAMH training of PHC providers increased the identification and reporting of children with mental health problems at the primary care level [53]. Given that a large portion of the population needs mental health services at community and primary care levels [10, 28], it is essential to find strategies to integrate CAMH at the PHC level and to reactivate the school mental health programme in Khartoum. The school mental health programme may represent a unique opportunity for detection, prevention, and treatment of different childhood mental health problems, given that it is based in the community, and could be integrated with primary health care levels to achieve accessibility and effective utilization of mental health services, while also increasing awareness of CAMH services [53]. The study could not identify other community platforms that might be used for mental health promotion/prevention.

The access to medications can be improved by providing the appropriate training to PHC providers in medication use and psychotherapy used for children and adolescents with mental health problems [54]. The current study revealed that psychostimulant medications were available only in the three National Medical Supply Fund pharmacies. Reviewing the list of essential medicine and improving the accessibility of PHC providers to medications used for children and adolescents with mental health problems and making them available would be an important next step.

It is important to identify CAMH professionals (numbers, professions, geographical distribution, and their level of training) suitable to work in facilities that provide care for children and adolescents with mental health needs in Khartoum. Given that there may never be a large specialist child & adolescent mental health workforce in Khartoum or in Sudan, it will be important to establish training programmes in child & adolescent mental health for all professionals working at all levels of healthcare and in other sectors such as education or social care who provide services for children and adolescents with mental health problems. These should include short courses and regular refresher trainings to increase staff competency, knowledge and confidence in the identification and management of the broad range of CAMH conditions. Bearing in mind the limited number of trained professionals working in CAMH services in LMICs, as pointed out by Simelane and de Vries [8], task-sharing with non-specialists and finding innovative strategies to coordinate and use the available workforce may represent the most realistic and immediate opportunity to strengthen the CAMH workforce in Sudan. Task-sharing may involve a clear job description for the CAMH professionals with appropriate delegation depending on their level of training. Furthermore, it has been emphasized that improving the training on CAMH to primary healthcare providers is essential for delivering CAMH services in LMICs. Different studies recommended that training should include aspects of child development, interviewing techniques, understanding of risk factors, recognition of child psychiatric disorders, evidence-based interventions, and helping parents respond effectively to the child’s emotional needs [55, 56]. This kind of training has shown good outcomes in CAMH [55]. It is also important to train all health workers who work with children and adolescents to be equipped to detect child abuse and to include mental health in the assessment of affected children. Another important goal in Sudan should be research capacity-building, as research training was indeed a major gap in Sudan, to ensure a new and well-trained generation of clinical CAMH researchers in Sudan and the Eastern Mediterranean Region.

Information about public education about CAMH in Khartoum was limited, and no data could be found on public perceptions of CAMH. This is an important gap to address, perhaps by increasing awareness about CAMH problems. This may increase the likelihood of help-seeking behaviour from young people and their families to access CAMH services [7, 8, 18]. In addition, studies have reported a lack of coordination of CAMH services with other child-care sectors with most LMICs (63%) having only a few schools providing CAMH promotion and prevention activities. Only 1% of schools in these LMICs had one or more mental health professionals as part of their staff [55]. The school mental health system identified in this situational analysis presents a very powerful potential resource and system, and efforts to strengthen or re-establish links between health and education would be a crucial next step in Sudan.

It is essential to have an organized health information system that gathers and provides key information and data on CAMH to strengthen the systems and services and allow monitoring of progress among countries [30]. Despite the data reported by the facilities that provide services for children and adolescents with mental health problems being variable and inconsistent, our situational analysis identified a potential information system but it seemed that the system had not been used to its full potential and that no CAMH specific data variables had been included in it to date. CAMH-specific items may include the sociodemographic characteristics of children and adolescents with mental health problems, frequency of visits, together with either ICD or DSM diagnoses. This will improve the quality of mental healthcare records and help periodic reviews of services and the needs before making appropriate modifications [2].

Our search for research output on CAMH in Sudan or from Sudan yielded 11 publications over 10 years, most of them in local journals. We have to conclude that CAMH research in Sudan is highly limited. National community-based epidemiological studies on the prevalence of different childhood mental health problems may be a very important contribution to understanding the landscape of CAMH needs in the country. Another important goal in Sudan should be research capacity-building, to ensure a new and well-trained generation of clinical CAMH researchers in Sudan and the Eastern Mediterranean Region. Rahman and colleagues proposed three broad targets for research in CAMH: (1) epidemiology of mental health problems and their risk factors, (2) comparative need assessment, and (3) corporate need analysis [56]. We propose that an essential component of research capacity building in Sudan should be efforts to build collaborative research in partnership with Government and across stakeholder groups (e.g. education, social care) to ensure that there is a joint agenda for research activities in the country [8]. It is also important to note that partnerships and collaboration between CAMH professionals from Sudan, other countries in the region (e.g., South Africa, Ethiopia, and Tunisia), and high-income countries could benefit CAMH services, research, and sharing of knowledge and experiences [56].

### Strengths and limitations of the study

This study was, to our knowledge, the first-ever systematic investigation of CAMH services and systems in Sudan and therefore represents baseline data that can be used to develop a joined-up plan for CAMH service strengthening in Khartoum State. We hope that the study will also provide a model for the examination of CAMH services and systems in other states of Sudan and other low-income countries. The most significant limitation of the study was the limited availability of data and documents that could be used in the situational analysis, hence the need for direct interaction with the Ministry of Health departments and relevant institutions to identify relevant documents. This was very much in keeping with our expectations, but still presented a stark reminder of the challenges in many LMICs to find appropriate and relevant data to map CAMH. For example, the situational analysis performed by Mokitimi and colleagues [7] in the Western Cape Province of South Africa (typically thought of as a well-resourced country and province) showed the many knowledge and data gaps that their study encountered. We acknowledge that this desktop situational analysis was performed during the COVID-19 pandemic and that findings reported here a) did not focus on the impact of the pandemic or b) had access to any data that could have commented on the impact of the pandemic on the CAMH system. However, as seen elsewhere around the globe we anticipate that the consequences of the COVID-19 pandemic were likely to have increased awareness of and need for access to mental health services and systems for young people, thus increasing the need for action in CAMH services and systems. In addition, the unstable political situation and the current ongoing armed-conflict in Sudan (and mainly in Khartoum) will exacerbate the CAMHSS challenges, and lead to less financing, disruption of the existed limted services, and shortages of trained mental health professionals working with children and adolescents [57]. We also acknowledge that a ‘desktop’ situational analysis represented only one component of comprehensive and multi-level situational analysis [6]. It would, therefore, be important to include the perspectives of senior stakeholders, service providers, and users as part of the next steps in research. As outlined in the introduction, the scope of this manuscript was very limited but has hopefully provided a helpful first step towards this broader goal.

In their broad-ranging *Lancet* Commission report on high-quality health systems in the time of the sustainable development goals era, Kruk and colleagues proposed the need for a ‘revolution’ to improve the quality of health systems. Even though we are completely aligned with the principle of improving quality of healthcare in low-income countries [58], many fundamental questions remain about the feasibility and appropriateness of health systems models, research and conceptual approaches that, almost exclusively, have been generated outside low-income countries (most of which are located in Africa, are politically unstable and war-torn). Perhaps it is particularly in low-income countries that truly ‘revolutionary’ models to provide child & adolescent mental health services are most needed.

## Conclusions

This situational analysis represents an important milestone in CAMH services and system development in Sudan by describing strengths and gaps that need to be addressed. CAMH policy and legislation need to be prioritized as they lay the foundation for the CAMH system and services planning and development. The study also highlighted the importance of collaboration between health sectors at different healthcare levels. This can be achieved by providing appropriate professional training in CAMH to different mental health professionals who work in facilities that provide services for children and adolescents with mental health disorders. Moreover, supporting and strengthening CAMH at primary care and community levels (e.g., the School Mental Health Programme) can help identify and manage children at risk for mental health difficulties, increase community awareness, and reduce stigma. The COVID-19 pandemic is likely to have increased the need for CAMHSS and this would be an important area for future research. The main goal for CAMH in Khartoum is to upgrade the existing facilities and resources, train the workforce, emphasize research, and extend collaboration while reducing stigma and barriers to CAMH.

## Data Availability

The situational analysis was based on publicly-available data. Any data not in the public domain can be requested from the authors.

## Abbreviations

CAMH: Child and Adolescent Mental Health
EMR: East Mediterranean Region
FMOH: Federal Ministry of Health
LMICs: Low- and Middle-Income Countries
PHC: Primary Health Care
UNDESA: United Nations Department of Economic and Social Affairs
UNFPA: United Nations Population Fund
UNICEF: United Nations Children’s Fund
WHO: World Health Organization
WHO-AIMS: World Health Organization Assessment Instrument for Mental Health Systems
